# Handling missing data in variational autoencoder based item response theory

**DOI:** 10.1111/bmsp.12363

**Published:** 2024-10-26

**Authors:** Karel Veldkamp, Raoul Grasman, Dylan Molenaar

**Affiliations:** ^1^ Department of Psychology University of Amsterdam Amsterdam The Netherlands

**Keywords:** missing data, multidimensional item response theory, variational autoencoders

## Abstract

Recently Variational Autoencoders (VAEs) have been proposed as a method to estimate high dimensional Item Response Theory (IRT) models on large datasets. Although these improve the efficiency of estimation drastically compared to traditional methods, they have no natural way to deal with missing values. In this paper, we adapt three existing methods from the VAE literature to the IRT setting and propose one new method. We compare the performance of the different VAE‐based methods to each other and to marginal maximum likelihood estimation for increasing levels of missing data in a simulation study for both three‐ and ten‐dimensional IRT models. Additionally, we demonstrate the use of the VAE‐based models on an existing algebra test dataset. Results confirm that VAE‐based methods are a time‐efficient alternative to marginal maximum likelihood, but that a larger number of importance‐weighted samples are needed when the proportion of missing values is large.

## 1 INTRODUCTION

Item response theory (IRT) is a popular latent variable framework in educational testing and psychology (Lord & Novick, 1968). It is used to estimate scores on latent variables based on a set of categorical item responses. This can be either a single latent variable in traditional IRT or multiple latent variables in multidimenisonal item response theory (MIRT; Reckase, 2009). Applications of these models include large‐scale online educational testing (Klinkenberg et al., 2011; Meyer & Zhu, 2013) and clinical assessment (Thomas, 2019) and often involve large datasets and high‐dimensional latent variables.

The classical way to estimate MIRT models is with marginal maximum likelihood (MML) using the expectation maximisation (EM) algorithm (Bock & Aitkin, 1981). This involves maximizing the marginal likelihood, in which the latent variables are integrated out. The dimensionality of this integral depends on the number of latent variables in the IRT model, making traditional Gauss–Hermite quadrature approximations computationally expensive for higher‐dimensional IRT models (e.g., Wood et al., 2002). Using adaptive quadrature can reduce the number of quadrature points needed per dimension (Schilling & Bock, 2005), but the number of quadrature points still grows exponentially with the number of dimensions.

Contemporary research has focused on various ways to approximate the integral in the marginal likelihood efficiently, such as Markov Chain Monte Carlo (MCMC) methods (e.g., Edwards, 2010) or stochastic approximation (e.g., Cai, 2010; von Davier & Sinharay, 2010). And both MCMC methods (Zhang et al., 2020) and stochastic approximation methods (Oka et al., 2024; Zhang & Chen, 2022) are still an active area of research. Although these methods substantially improve the efficiency of estimation of multi‐dimensional models, the exponential growth of complexity as the latent dimensionality increases still limits the use of these methods in high‐dimensional applications.

Yet another approach that avoids the computation of the marginal likelihood is the use of variational inference (Blei et al., 2017). Instead of maximizing the marginal likelihood, these methods approximate the posterior distribution of the latent variables with a different distribution and maximize the resulting lower bound on the marginal log‐likelihood. Recently, Cho et al. (2021) successfully used these methods to estimate up to seven‐dimensional IRT models. Ma et al. (2023) showed that such variational estimation of MIRT parameters might lead to slightly biased discrimination estimates, but they showed that taking multiple importance‐weighted samples from the approximate posterior could mitigate this issue, at the cost of slightly higher computational time. The computational efficiency of these methods can be further improved using amortised variational inference (AVI) (Zhang et al., 2018). Rather than optimizing posterior parameters directly, in AVI a function is estimated that predicts the posterior parameters from the observed data by maximizing the same lower bound. By equating the parameters of this function across all observations, AVI dramatically improves efficiency for large datasets. The most canonical form of AVI is the variational autoencoder (VAE), which uses a feedforward neural network (FNN) (Svozil et al., 1997) as the function estimating the posterior parameters. This is a class of models used in the machine learning community that serve as universal function approximators (Hornik et al., 1989). Curi et al. (2019). used this VAE approach to estimate high‐dimensional MIRT models on large datasets and show that the estimation is on par with traditional MML while being up to 40 times faster in estimation. Later research expands on this idea, showing that a generalization of the lower bound using importance sampling can approximate the true marginal likelihood arbitrarily well (Burda et al., 2015; Urban & Bauer, 2021). Urban and Bauer (2021). used this adapted lower bound to estimate MIRT models with up to 10 dimensions. Moreover, research has generalized this approach to various applications, such as correlated posteriors (Converse et al., 2021) and three‐ and four‐parameter logistic models (Liu et al., 2022).

Despite being a promising framework for estimating high‐dimensional MIRT models, a drawback of the VAE approach is that dealing with missing data is not straightforward. Where traditional methods draw strength from being full information maximum likelihood procedures in which missing data can easily be dealt with, a FNN cannot handle missing input values naturally. There are various approaches to missing data in the general literature on VAEs, but only two pragmatic and potentially suboptimal methods have been adapted to the MIRT context (Liu et al., 2022; Montecino, 2023). In this study, we adapt three missing data techniques from the VAE literature to VAE‐based MIRT modelling and propose one new technique. In a simulation study in which we vary the degree of missing data, the performances of the four approaches are systematically compared to each other and to full information MML using Metropolis‐Hastings Robbins‐Monro (MHRM) estimation, which is a state‐of‐the‐art MML estimation procedure (Cai, 2010).

In this paper we focus on estimation based on (a lower bound to) the marginal likelihood, as, arguably, marginal likelihood–based estimation is currently the most popular in IRT. However, we note that other estimation procedures are not based on the (marginal) likelihood, which can give a fast alternative to high‐dimensional MML estimation, such as constrained (Chen et al., 2019) and regularized (Bergner et al., 2022) joint maximum likelihood and‐least squares estimation (Browne, 1974; Muthén, 1984).

The outline of this paper is as follows. First, we briefly discuss FNNs, as these are important tools used in VAE‐based MIRT. We then formally introduce MIRT and show the challenges associated with MML for high‐dimensional IRT models. Next, we discuss variational inference and VAEs for MIRT models and derive different approaches to dealing with missing data in this paradigm. We present a thorough parameter recovery simulation study comparing the different missing data techniques to each other and to MML and a short second simulation to demonstrate that the proposed models can deal with correlated latent variables. Finally, we demonstrate the use of the methods on a real dataset pertaining to a large‐scale algebra test and end with our general conclusion and recommendations on how to deal with missing data in VAE–based MIRT modelling.

## 2 FEED FORWARD NEURAL NETWORKS

FNNs are a popular class of methods in the field of machine learning. They map a p‐dimensional vector of predictor variables x to an o‐dimensional vector of outcome variables y. As the name suggests, a FNN consists of multiple layers, where the values of variables in the next layer depend on the values of the previous layer:

(1) $$h_{i+1}=f_{i}(W_{i}h_{i}+b_{i}),i=0,1,\text{…}I,$$

where $W_{i}$ is a matrix of weight parameters for layer i, $b_{i}$ is a vector of bias parameters for layer i, and $f_{i}$ is a non‐linear function called an activation function. $h_{i}$ denotes the $n_{i}$‐dimensional representation vector in layer i, where $n_{i}$ is the dimensionality of the layer. Note that for convenience $h_{0}$ denotes the N‐dimensional vector of original observations x, and $h_{I}$ denotes the outcome predictions $\hat{y}$. The intermediate representations $h_{i},0<i<I$, are generally referred to as hidden layer activations, and the individual elements in $h_{i}$ are referred to as hidden nodes. Conceptually, a FNN consists of multiple linear transformations, each followed by a non‐linear function. Hornik et al. (1989) has shown that theoretically, FNNs with just a single hidden layer can approximate any Borel measurable function to any degree of accuracy, given enough hidden nodes, making FNNs a form of universal function approximators.

The parameters of a FNN are generally estimated by numerical optimization of a loss function $\Lambda (y,\hat{y})$, such as the log‐likelihood, using gradient descent. Modern implementations use Stochastic gradient descent (SGD) (Amari, 1993). The core idea of SGD is to iteratively update parameter values by partitioning the dataset into random disjoint subsets ${\left\{x_{b}\right\}}_{b=1}^{B}$ (called mini‐batches), computing the predicted values ${\left\{{\hat{y}}_{b}\right\}}_{b=1}^{B}$, and updating the parameters by adding the gradients of the loss function with respect to the weights and biases multiplied by a learning rate, which is a hyperparameter that determines the size of the updates. This process is repeated until some stopping criterion is reached. The partitioning of the dataset allows the estimation process to be faster, while also making it less sensitive to local minima. The specific algorithm used in this study is the AMSGrad algorithm (Reddi et al., 2019), which is currently one of the most popular adaptations of SGD for FNNs. It makes use of an adaptive learning rate, allowing for bigger updates when gradients are small, accelerating convergence, and smaller updates when gradients are big, preventing overshooting. We refer the reader to Reddi et al. (2019) for a detailed discussion of AMSGrad.

## 3 MIRT

The Multidimensional 2PL (M2PL) model is the most common MIRT model (McKinley & Reckase, 1983) due to its relation to item factor analysis (Takane & De Leeuw, 1987; Wirth & Edwards, 2007). In this model, the probability of a correct response is modelled as

(2) $$P(X_{ij}=1|\theta_{j})=\frac{1}{1+\exp(-a_{j}^{T}\theta_{i}-b_{j})},$$

where $X_{ij}$ is a random variable representing the response of participant i on item j, $\theta_{i}$ is the latent variable vector for person i, $a_{j}$ is the item slope vector for item j, and $b_{j}$ is the item intercept for item j. The latent variable parameter vectors represent scores on the multidimensional latent ability constructs, whereas the item parameters represent the degree to which an item discriminates between different levels of each latent construct, as well as how likely an item is to receive a positive response generally. The marginal log‐likelihood for person i is given by

(3) $$\log P(X_{i}=x_{i}|\Omega )=\log\int \text{⋯}\int \overset{J}{\underset{j=1}{\prod }}P(X_{ij}=x_{ij}|\theta ;\Omega_{j})p(\theta )d\theta_{1}d\theta_{2}\text{⋯}d\theta_{D},$$

where $X_{i}$ is a vector of random variables representing the binary item responses for subject i with elements $X_{ij}$, $\Omega_{j}=\{a_{j},b_{j}\}\in \Omega$ is the set of item parameters for item j, p(θ) is the prior probability of the ability estimate θ, and D is the number of latent dimensions. Some elements of the item discrimination parameters $a_{j},j=1,\text{…},J$, must be fixed at zero to avoid rotational indeterminacy. The complete marginal log‐likelihood is simply the sum over all participants.

### 3.1 Variational inference

As discussed in the introduction, variational inference provides an alternative way to estimate MIRT parameters, avoiding the need for this computationally expensive integral that needs to be approximated in MML. In variational inference, rather than optimizing the marginal likelihood directly, the posterior density $p(\theta_{i}|x_{i})$, for which there is generally no closed‐form expression, is approximated by another density $\hat{q}(\theta_{i}|x_{i})$ such that the Kullback–Leibler (KL) divergence is minimized over a (parameterized) family ℱ of proposal distributions:

(4) $$\hat{q}(\theta_{i}\;|\;x_{i})=\underset{q(\theta |x)\in ℱ}{\text{argmin}}\text{KL}[q(\theta_{i}|x_{i})||p(\theta_{i}|x_{i})]$$

(Blei et al., 2017). Since this KL divergence in this expression requires the true posterior, we cannot optimize it directly. However, it can be shown that minimizing this KL divergence is equivalent to maximizing the evidence lower bound (ELBO), which is defined as

(5) $$\text{ELBO}={\mathrm{34𝔼}}_{\hat{q}}[\log p(x_{i}|\theta_{i})]-\text{KL}[q(\theta_{i}|x_{i})||p(\theta_{i})],$$

where $p(\theta_{i})$ is the prior density over $\theta_{i}$ (i.e., the population density of the latent ability scores). The ELBO derives its name from the fact that it is a lower bound to the marginal log‐likelihood. Specifically, the marginal log‐likelihood can be expressed as

(6) $$\log p(x_{i})=\text{KL}[\hat{q}(\theta_{i}|x_{i})||p(\theta_{i}|x_{i})]+\text{ELBO}.$$

This indicates that the ELBO approaches the marginal log‐likelihood as the approximation distribution approaches the true posterior. Choosing a flexible family ℱ for q allows this divergence to be minimized as much as possible. We will address the choice for q in what follows. Variational inference has successfully been used as a method of estimating MIRT parameters (Cho et al., 2021; Ma et al., 2023).

### 3.2 Amortized variational inference

Where traditional variational inference optimizes the parameters of the approximate posterior directly, amortized variational inference estimates a mapping from the observed data to the posterior parameters. This means that instead of estimating a separate posterior distribution for the latent ability of each respondent, we estimate the parameters of a so‐called inference model that predicts the posterior distribution of the latent ability parameters from a pattern of item responses. This function is generally chosen to be approximated with a FNN (Converse et al., 2021; Curi et al., 2019; Liu et al., 2022; Urban & Bauer, 2021; Wu et al., 2020), and the parameters are shared across observations. As a result, if the posterior distribution of the latent variables is assumed to be a multivariate normal distribution, the latent variables $\theta_{i}$ are modelled by

(7) $$\theta_{i}∼MVN(\mu_{i},\text{diag}(\sigma_{i})),\;\text{with}\;(\mu_{i},\sigma_{i})={\text{FNN}}_{\phi }(x_{i}),$$

where ϕ are the parameters of the inference model. The full model is a VAE (Kingma & Welling, 2013) in which the parameters are estimated iteratively by taking a sample ${\hat{\theta }}_{i}$ from the multivariate normal (cf. Equation 7), with the FNN evaluated at the current best estimates of $\hat{\phi }$. Then, $\hat{\Omega }$ and $\hat{\phi }$ are jointly updated to maximize the ELBO with respect to these parameters. That is, the new estimates are

(8) $$\hat{\phi },\hat{\Omega }=\underset{\phi ,\Omega }{\text{argmax}}\mathrm{34𝔼}[\log p(x_{i}|{\hat{\theta }}_{i};\Omega )]-KL[q(\theta_{i}|x_{i};\phi )||p(\theta_{i})],$$

where the approximate posterior q(.) is given by Equation (7). In the simplest case, the population density $p(\theta_{i})$ is fixed to a standard multivariate normal density, but it is straightforward to accommodate correlated latent abilities in this prior. In what follows we discuss how this assumption of a normal approximate posterior can be relaxed.

### 3.3 Importance weighted amortized variational inference

As discussed above, VAEs generally assume that the posterior distribution of $\theta_{i}$ is normal and highly factorized (e.g., multivariate normal with diagonal covariance matrix). These constraints on the posterior can cause the ELBO to severely underestimate the true marginal log‐likelihood. Recent research has focused on various ways of tightening this lower bound (Burda et al., 2015; Rezende & Mohamed, 2015). One of these approaches, proposed by Burda et al. (2015), is the importance‐weighted variational autoencoder (IWVAE). This model has the same structure as a regular VAE, but instead of taking a single sample of the approximate posterior, multiple samples are taken in order to calculate an importance‐weighted estimate of the log‐likelihood:

(9) $$\text{IW‐ELBO}={\mathrm{34𝔼}}_{1:k}\left[\log\frac{1}{K}\overset{K}{\underset{k=1}{\sum }}\frac{p(x_{i},{\hat{\theta }}_{i}^{\left(k\right)})}{q({\hat{\theta }}_{i}^{\left(k\right)}|x_{i})}\right],$$

where K is the number of importance samples and the term inside the sum are the unnormalized importance samples for the joint distribution. When K=1, the importance‐weighted evidence lower bound (IW‐ELBO) is equivalent to the regular ELBO in Equation (5). Using multiple importance‐weighted samples from the approximate posterior tightens the lower bound to the marginal log‐likelihood. Burda et al. (2015) show that the IW‐ELBO approaches $\log p(x_{i})$ as K goes to infinity, making Equation (9) a MML estimator. In a later paper Cremer et al. (2017) show that by taking these multiple importance‐weighted samples from the approximate posterior, you are effectively using a more flexible mixture distribution as your approximate posterior, relaxing the assumption that the posterior must be a multivariate normal. They also demonstrate that you can compute expected a posteriori (EAP) ability estimates from this more flexible posterior using importance sampling. Specifically, you can sample from the approximate posterior by taking K samples from the multivariate normal distribution and taking a sample from these K samples using the importance weights. By repeating this process M times and taking the mean, a Monte Carlo estimate of the EAP ability can be obtained. The IWVAE was first used in the context of MIRT by Urban and Bauer (2021), who showed empirically that the IWVAE can provide accurate MIRT parameter estimates for up to 10 dimensions while being much faster than traditional EM algorithms.

### 3.4 The challenge of missing data

Although the amortization step allows IWVAEs to efficiently estimate complex latent variable models, it introduces a problem regarding missing data. Since the model now includes an inference model that predicts the parameters of the latent variable distribution based on an observed response pattern (Equation 7), this function needs to be capable of dealing with missing data. A standard FNN does not meet this requirement, since missing values make it impossible to calculate gradients with respect to the network parameters. Simply neglecting the missing values in $x_{i}$ will cause $\mu_{i}$ and $\sigma_{i}$ in Equation (7) to be on a different scale for respondents with different missing value patterns, which will greatly bias the results. Furthermore, list‐wise deletion, pair‐wise deletion, and mean imputation are not recommended in general for loss of statistical efficiency. Liu et al. (2022) employ an intuitive trick to handle missing data in VAEs. However, as will be discussed in what follows, this approach is suboptimal and potentially biases item parameter estimates, especially in cases where the majority of the data is missing. Thus, more work is needed to adapt VAE‐based IRT models to missing data. Below, we derive four explicit approaches to handling missing data in IWVAEs and provide a systematic comparison.

## 4 HANDLING MISSING DATA

### 4.1 Input drop‐out

An intuitive way of dealing with missing data is the so‐called *input‐dropout trick* (Liu et al., 2022; Nazabal et al., 2020). This trick consists of replacing the missing values in the input pattern with zeros and only calculating the loss of the VAE over the non‐missing datapoints. The new loss function for the VAE using the input‐dropout trick is

(10) $$\mathrm{ELB}O_{\mathrm{ID}}={\mathrm{34𝔼}}_{q(\theta_{i}|{\tilde{x}}_{i};\phi )}[\log p(x_{ij},j\in O_{i}|\theta_{i};\Omega )]-KL[q(\theta_{i}|{\tilde{x}}_{i};\phi )||p(\theta_{i})],$$

where ${\tilde{x}}_{i}$ is the response pattern with missing values replaced by zero, and $O_{i}$ is the set of indices of observed item responses. This is the method that was used in the context of MIRT by Liu et al. (2022).

A problem with the input‐dropout trick is that although the reconstruction loss is only calculated over the observed data points, the inference model has no way to distinguish between missing values and responses that are truly zero, making it harder to estimate the latent ability variables. This may be suboptimal especially in MIRT applications, as zero responses are frequent in binary response data.

### 4.2 Conditional VAEs

To mitigate the problem above, Collier et al. (2020) introduced the *conditional variational autoencoder (CVAE)*. In this approach, the loss function is equivalent to the input‐dropout loss ELBO_ID_ in Equation (10), but the inference network now takes both the response pattern and the mask of missing data as input:

(11) $$\theta_{i}∼N_{d}(\theta ;\mu_{i},\sigma_{i}),\;\text{with}\;(\mu_{i},\sigma_{i})={\text{FNN}}_{\phi }(x_{i},m_{i}).$$

Here $m_{i}$ is a binary *mask* vector that indicates which values are missing in $x_{i}$ and which are not, i.e., $m_{ij}=1$ if $j\in O_{i}$ and $m_{ij}=0$ if $j\notin O_{j}$. This additional input to the inference model allows it to differentiate between missing values and true zero responses.

This model has not been considered yet in the context of MIRT, and we consider the model as an alternative approach to handling missing data in this context.

### 4.3 Partial VAEs

A third approach to missing data consists of *partial variational autoencoders (PVAE)* (Ma et al., 2018). PVAEs were originally developed for the application of recommender systems, in which large fractions of missing data are common. They are based on neural networks for point net classification^1^ that can deal with variable input sizes (Qi et al., 2017). Although FNNs generally require a fixed input size for each respondent, PVAEs bypass this requirement by estimating a so‐called *embedding* vector for each item. An embedding vector in this context is an estimated vector of parameters. By combining the embeddings from all items with an observed response using a permutation‐invariant aggregation operation, unobserved items can be simply ignored in the hope that the estimated embeddings will still encode essential information about $\mu_{i}$ and $\sigma_{i}$.

(12) $$\theta_{i}∼N(\theta ;\mu_{i},\sigma_{i}),\;\text{with}\;(\mu_{i},\sigma_{i})={\text{FNN}}_{\phi }(g(\{e_{j}:j\in O_{i}\})).$$

Here, $e_{j}$ denotes the embedding for item j, and g is a well‐defined permutation‐invariant function that maps a set of points in embedding space to a single point in that same space, for example, the mean vector, or the vector of coordinate‐wise maxima. This function g allows for a variable amount of embeddings and returns an output vector of a fixed length. The FNN takes the output from g and computes the variational parameters as in a regular VAE. Alternatively, g can be extended to return a vector of descriptive statistics of the embeddings, such as the mean, standard deviation, or distribution quantiles, to yield a more detailed representation of the distribution of the embedding vectors.

### 4.4 Imputation VAE

Finally, we propose a new approach, which we will refer to as the *imputation variational autoencoder (IMVAE)*. In this model, we use the same input‐dropout loss function ELBO_ID_ in Equation (10), but rather than imputing the input with zeros, we impute the missing values with the expected values for the responses given the current IRT model parameter estimates. Specifically, we set

(13) $${\tilde{x}}_{i}=x_{i}⊙m_{i}+p(x_{i}|{\hat{\theta }}_{i};\hat{\Omega })⊙(1-m_{i}),$$

where ⊙ denotes the Hadamard products, 1 is a commensurate vector of ones, and ${\hat{\theta }}_{i}$ again is the vector of latent variable estimates for subject i based on the current estimates $\hat{\phi }$ of the inference model parameters. In the first iteration, ${\hat{\theta }}_{i}$ is set to the expected value of the prior. This adaptation still calculates the loss using only the observed data patterns but allows the inference model to make better estimates of the latent ability variables by using the current model parameters to estimate the most likely values of the missing data. Note that our proposed method is similar to an approach proposed by Montecino (2023, chapter 3).^2^ However, our approach differs in two ways: First, we use the actual predicted probabilities while Montecino (2023) rounded these probabilities to zero and one which distorts the stochastic properties of the responses. Additionally, Montecino (2023). used a regular VAE while we embed our approach in an IWVAE, which, as discussed above, will provide a closer approximation to the true posterior.

## 5 SIMULATION STUDY

We simulated datasets of 10,000 respondents from three‐dimensional (3D) and 10‐dimensional (10D) MIRT models. Latent variable values were sampled from a standard normal $\theta ∼N_{d}(0,I_{d})$. For simplicity, latent variables were uncorrelated. However, note that the flexibility of the approximate posterior introduced by taking multiple importance samples does actually allow for correlations between factors, which we will demonstrate in Section 7. Item slope parameters were sampled from a uniform distribution $a_{j}∼U(.5,2),j=1,\text{…},J$, and intercept parameters $b_{j}$ were set to equally spaced values between −2 and 2. Several slope parameters were set to zero. For the 3D model, we simulate 28 items and fix slope parameters to zero based on a factor configuration from the literature (Curi et al., 2019; da Silva et al., 2019). For the 10D model, we simulate 110 items based on our own configuration where each item depends on one or two latent factors. Both configurations are available on the GitHub page,^3^ along with all of the code necessary to reproduce our experiments or to apply our models to new data.

Missing values were introduced at random by deleting a proportion of observed responses. The proportion of missing data was varied to 10 equally spaced values between 0 and .75, inclusive. We ran 50 replications for each combination of dimensionality and missingness, resulting in 1000 unique datasets. We fit the input dropout variational autoencoder (IDVAE), CVAE, PVAE, and IMVAE to each dataset and estimate each model using one, five, and 25 importance weight samples, resulting in a 2×10×4×3 factorial design. We compare results to MML using state‐of‐the‐art MHRM (Cai, 2010) estimation as implemented in the R package *mirt* (Chalmers, 2012). We used the EAP ability estimates for the MML models, which were estimated using quasi‐Monte Carlo estimating in the 10D case. For the VAE‐based models we use the ability estimation procedure outlined in Section 3.2, which produces a Monte Carlo estimate of the expectation of the approximate posterior.

### 5.1 Implementation details

All our models are implemented in PyTorch (Paszke et al., 2019), and all code is publicly available on GitHub. Models were estimated using the AMSgrad algorithm using a learning rate of .005 and a batch size of 32. We stopped estimation when the loss did not decrease by more than $10^{-7}$ over 10 iterations.

We used a single hidden layer of 20 nodes for the IDVAE, CVAE, and IMVAE. In the PVAE, an embedding of length 12 was learned for each item. These embeddings were transformed to a vector of length 24 using a single‐layer FNN. We transform these item vectors to a fixed‐length person vector using a permutation‐invariant operation. Specifically, we computed the mean, median, standard deviation, and the 25th and 75th quartiles of each person's item distribution and concatenated the results, yielding a vector of length 120 for each person. The resulting person vectors were used as input for a regular VAE encoder with a single hidden layer of 24 nodes. We have also experimented with using a single permutation‐invariant function, but using a concatenation has provided better results in our experience.

## 6 RESULTS

**FIGURE 1:**
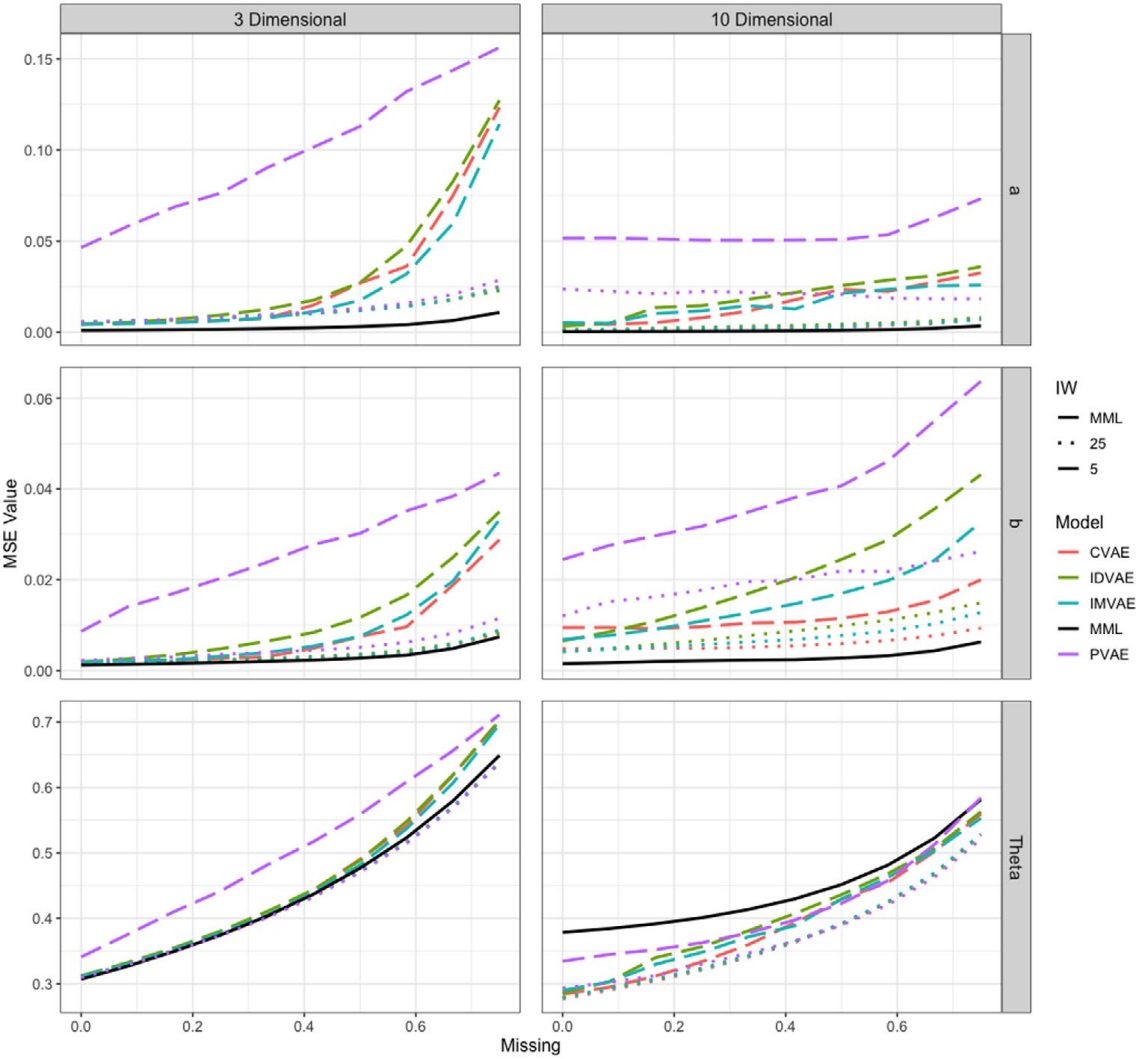
MSE of parameter estimates for increasing proportion of missing data. Note that results for models estimated using a single importance weight are omitted.

Figure 1 presents the average mean squared error (MSE) of the parameter estimates for the different models and different numbers of importance‐weighted samples as the proportion of missing data increases. Parameter estimates using a single importance sample were very poor and were left out of the figure to prevent obscuring the image. Overall we see that the accuracy of VAE‐based parameter estimates decreases rapidly as the proportion of missing data increases. However, as the amount of importance samples increases, the variational methods approach the accuracy of MML. This is in line with expectations, as increasing the number of samples tightens the bound to the marginal log‐likelihood. As more data are missing, a larger number of samples is needed to attain accurate parameter estimates. However, as becomes clear from Table 1 4, using VAE‐based methods with a large number of IW samples is still computationally much faster than MML in high‐dimensional applications. The complete results for the 3D and 10D models are available in Tables 2 and 3 respectively.

**TABLE 1:** Average runtime (minutes) of each model over 10 replications. IW is the number of importance‐weighted samples, and d is the dimensionality of the model. All models were estimated on a single CPU core of the Apple MacBook Pro M3.

Miss	Model	IW = 1		IW = 5		IW = 25	
		d=3	d=10	d=3	d=10	d=3	d=10
.00	cvae	.55	.46	.47	.41	.66	.90
	idvae	.53	.42	.53	.52	.53	.96
	imvae	.54	.42	.59	.65	.52	.86
	pvae	1.67	1.46	.98	3.88	4.26	3.84
	mirt	4.27	19.05	–	–	–	–
.25	cvae	.45	.55	.50	.53	.61	.94
	idvae	.51	.48	.47	.49	.55	1.00
	imvae	.51	.49	.43	.50	.60	.95
	pvae	1.18	1.48	1.03	2.11	3.47	3.57
	mirt	4.40	20.34	–	–	–	–
.75	cvae	.39	.48	.45	.46	.73	.91
	idvae	.40	.50	.54	.35	.54	1.09
	imvae	.42	.48	.57	.42	.62	.89
	pvae	1.02	1.36	1.27	1.67	2.15	2.17
	mirt	4.49	11.54	–	–	–	–

**TABLE 2:** Simulation study results for 3D models. m denotes the proportion of missing data, p the parameter being estimated, and $\sigma^{2}$ the average variance of the parameter estimates. Note that MML is arbitrarily placed under IW = 1 to keep the table concise, but it does not make use of importance weighting. For the sake of brevity, the table only contains three levels of missing data. The complete table is available on GitHub.

p	m	Model	IW = 1			IW = 5			IW = 25		
			$\sigma^{2}$	$bias^{2}$	mse	$\sigma^{2}$	$bias^{2}$	mse	$\sigma^{2}$	$bias^{2}$	mse
a	.00	cvae	.0036	.1524	.1560	.0037	.0591	.0628	.0001	.0256	.0258
		idvae	.0129	.2059	.2188	.0024	.0730	.0754	.0001	.0258	.0259
		ivae	.0231	.2236	.2467	.0022	.0737	.0759	.0001	.0258	.0260
		pvae	.0071	.4332	.4403	.0005	.1326	.1331	.0002	.0354	.0356
		mirt	.0009	.0000	.0009	–	–	–	–	–	–
	.25	cvae	.0140	.2853	.2993	.0015	.1108	.1123	.0021	.0380	.0401
		idvae	.0150	.3967	.4117	.0012	.1134	.1146	.0019	.0375	.0395
		ivae	.0176	.3994	.4170	.0013	.1116	.1129	.0014	.0368	.0381
		pvae	.0013	.6304	.6317	.0015	.1540	.1555	.0007	.0491	.0498
		mirt	.0023	.0000	.0024	–	–	–	–	–	–
	.75	cvae	.0008	.6378	.6386	.0054	.5154	.5208	.0062	.2039	.2102
		idvae	.0007	.6381	.6389	.0070	.4640	.4710	.0074	.1876	.1950
		ivae	.0007	.6371	.6379	.0065	.4748	.4812	.0040	.1754	.1795
		pvae	.0008	.6376	.6384	.0048	.5062	.5111	.0058	.1817	.1875
		mirt	.0859	.0690	.1549	–	–	–	–	–	–
b	.00	cvae	.0001	.0555	.0556	.0002	.0435	.0436	.0002	.0335	.0336
		idvae	.0004	.0652	.0656	.0001	.0462	.0462	.0001	.0339	.0341
		ivae	.0008	.0685	.0693	.0001	.0463	.0464	.0001	.0341	.0342
		pvae	.0005	.0828	.0833	.0001	.0491	.0492	.0001	.0371	.0372
		mirt	.0010	.0000	.0011	–	–	–	–	–	–
	.25	cvae	.0005	.0798	.0803	.0001	.0564	.0565	.0002	.0454	.0455
		idvae	.0004	.0945	.0949	.0001	.0580	.0581	.0002	.0456	.0458
		ivae	.0003	.0949	.0952	.0001	.0578	.0579	.0001	.0457	.0459
		pvae	.0000	.1025	.1025	.0001	.0613	.0614	.0001	.0482	.0483
		mirt	.0022	.0000	.0022	–	–	–	–	–	–
	.75	cvae	.0000	.1208	.1208	.0000	.1196	.1196	.0001	.1015	.1016
		idvae	.0000	.1208	.1208	.0000	.1184	.1184	.0001	.0992	.0993
		ivae	.0000	.1208	.1208	.0000	.1186	.1186	.0001	.0980	.0981
		pvae	.0000	.1209	.1209	.0000	.1194	.1194	.0001	.0984	.0985
		mirt	.0341	.0182	.0523	–	–	–	–	–	–
θ	.00	cvae	.0147	.4927	.5074	.0232	.3610	.3842	.0083	.3363	.3446
		idvae	.0456	.5239	.5696	.0211	.3903	.4115	.0084	.3368	.3453
		ivae	.0645	.5314	.5959	.0202	.3901	.4103	.0085	.3367	.3452
		pvae	.0619	.7426	.8045	.0163	.4897	.5061	.0099	.3418	.3518
		mirt	.1990	.1369	.3359	–	–	–	–	–	–
	.25	cvae	.0432	.6278	.6709	.0163	.5048	.5211	.0144	.4098	.4243
		idvae	.0516	.7057	.7573	.0155	.5145	.5300	.0139	.4103	.4242
		ivae	.0574	.6983	.7557	.0151	.5125	.5277	.0123	.4094	.4217
		pvae	.0110	.9799	.9908	.0211	.5465	.5675	.0123	.4209	.4331
		mirt	.2222	.2526	.4747	–	–	–	–	–	–
	.75	cvae	.0060	.9952	1.0012	.0275	.8946	.9221	.0224	.7492	.7717
		idvae	.0021	.9948	.9968	.0279	.8636	.8915	.0225	.7443	.7668
		ivae	.0024	.9960	.9984	.0274	.8738	.9013	.0196	.7389	.7585
		pvae	.0055	.9975	1.0030	.0261	.8924	.9184	.0211	.7412	.7623
		mirt	.0770	.8328	.9098	–	–	–	–	–	–

**TABLE 3:** Simulation study results for 10D model. m denotes proportion of missing data, p parameter being estimated, and $\sigma^{2}$ variance of parameter estimates. Note that MML is arbitrarily placed under IW = 1 to keep the table concise, but it does not make use of importance weighting. For the sake of brevity, the table only contains three levels of missing data. The complete table is available on GitHub.

p	m	Model	IW = 1			IW = 5			IW = 25		
			$\sigma^{2}$	$bias^{2}$	mse	$\sigma^{2}$	$bias^{2}$	mse	$\sigma^{2}$	$bias^{2}$	mse
a	.00	cvae	.0158	.0647	.0805	.0016	.0249	.0266	.0001	.0165	.0167
		idvae	.0141	.0711	.0852	.0018	.0243	.0261	.0003	.0170	.0173
		ivae	.0130	.0724	.0854	.0021	.0245	.0266	.0002	.0170	.0172
		pvae	.0039	.2680	.2720	.0024	.1024	.1049	.0009	.0559	.0568
		mirt	.0004	.0001	.0004	–	–	–	–	–	–
	.25	cvae	.0143	.1006	.1148	.0043	.0374	.0418	.0009	.0213	.0222
		idvae	.0141	.1409	.1549	.0053	.0393	.0445	.0004	.0212	.0216
		ivae	.0169	.1454	.1623	.0041	.0353	.0394	.0007	.0202	.0209
		pvae	.0002	.3095	.3097	.0038	.1150	.1188	.0013	.0593	.0606
		mirt	.0009	.0000	.0009	–	–	–	–	–	–
	.75	cvae	.0003	.3100	.3103	.0051	.1932	.1983	.0059	.0770	.0830
		idvae	.0003	.3098	.3101	.0074	.1645	.1719	.0058	.0594	.0651
		ivae	.0003	.3101	.3104	.0052	.1923	.1976	.0068	.0711	.0779
		pvae	.0003	.3102	.3105	.0044	.2057	.2101	.0055	.0826	.0881
		mirt	.0393	.0034	.0427	–	–	–	–	–	–
b	.00	cvae	.0014	.1665	.1679	.0003	.2119	.2122	.0000	.2193	.2193
		idvae	.0010	.1707	.1717	.0001	.2141	.2142	.0000	.2194	.2194
		ivae	.0010	.1677	.1687	.0002	.2139	.2141	.0001	.2187	.2187
		pvae	.0001	.1451	.1453	.0002	.1658	.1660	.0001	.1966	.1967
		mirt	.0011	.0003	.0014	–	–	–	–	–	–
	.25	cvae	.0003	.1342	.1345	.0004	.1691	.1695	.0001	.1860	.1861
		idvae	.0005	.1421	.1426	.0002	.1796	.1798	.0001	.1911	.1911
		ivae	.0005	.1429	.1434	.0002	.1783	.1785	.0001	.1907	.1907
		pvae	.0000	.1528	.1528	.0002	.1526	.1528	.0001	.1766	.1767
		mirt	.0020	.0009	.0028	–	–	–	–	–	–
	.75	cvae	.0000	.1713	.1713	.0000	.1653	.1654	.0001	.1495	.1496
		idvae	.0000	.1713	.1713	.0001	.1616	.1617	.0001	.1491	.1492
		ivae	.0000	.1713	.1713	.0000	.1656	.1657	.0001	.1503	.1504
		pvae	.0000	.1713	.1713	.0000	.1662	.1663	.0001	.1509	.1510
		mirt	.0454	.0035	.0489	–	–	–	–	–	–
θ	.00	cvae	.1169	.3778	.4947	.0206	.3279	.3485	.0092	.3184	.3276
		idvae	.1237	.3992	.5229	.0278	.3288	.3566	.0133	.3194	.3327
		ivae	.1202	.4083	.5284	.0292	.3301	.3593	.0132	.3195	.3327
		pvae	.1113	.8111	.9225	.0654	.4859	.5513	.0297	.3939	.4236
		mirt	.1967	.1809	.3777	–	–	–	–	–	–
	.25	cvae	.1166	.4746	.5912	.0349	.3925	.4274	.0138	.3700	.3838
		idvae	.1335	.5466	.6801	.0450	.4066	.4516	.0150	.3720	.3870
		ivae	.1578	.5336	.6914	.0382	.3956	.4338	.0161	.3698	.3859
		pvae	.0075	.9932	1.0006	.0648	.5339	.5986	.0284	.4383	.4667
		mirt	.2049	.2304	.4353	–	–	–	–	–	–
	.75	cvae	.0052	.9959	1.0011	.0419	.7497	.7916	.0316	.6201	.6518
		idvae	.0023	.9950	.9973	.0483	.7107	.7590	.0294	.6080	.6374
		ivae	.0037	.9958	.9995	.0409	.7528	.7937	.0335	.6160	.6495
		pvae	.0063	.9966	1.0029	.0362	.7852	.8214	.0298	.6349	.6647
		mirt	.1271	.7221	.8493	–	–	–	–	–	–

The relative performance of variational methods is generally consistent across conditions. The CVAE parameter estimates have the lowest MSE. The CVAE and IMVAE are very close with respect to their item parameter estimates, but the extra information provided to the inference model appears to allow the CVAE to estimate ability parameters with more precision. Both the CVAE and IMVAE are consistently more accurate than the IDVAE, which is in line with expectations, as these two methods can be conceived of as improvements upon the IDVAE. The PVAE is clearly the least effective method to estimate MIRT parameters, performing substantially worse than all other methods across conditions. Somewhat surprisingly, the variational method outperforms MML in terms of the ability estimates, especially in the high‐dimensional case. This might be due to the fact that we use quasi‐Monte Carlo integration to approximate the posterior theta distribution for the MML model.

## 7 CORRELATED LATENT VARIABLES

Although we have so far only considered data obtained with uncorrelated latent factors in order to keep the simulation design simple, this is not a requirement for the VAE‐based approach: Because the IWAE encoder can be used to sample from an approximation of the posterior that approaches the true posterior as K→∞ (Cremer et al., 2017), we can obtain an estimate of the covariance matrix of the latent factors from samples from this approximate posterior by means of the following expression:

$$\hat{\Sigma }=\frac{1}{NM}\overset{N}{\underset{i=1}{\sum }}\overset{M}{\underset{m=1}{\sum }}\eta_{im}\eta_{im}^{\prime },\;\eta_{im}∼f(\theta |X_{1}=x_{i1},\text{…},X_{J}=x_{iJ};\hat{\Omega },\hat{\phi }).$$

Here the $\eta_{im}$ are samples from the approximate posterior $f(\theta |X_{1},\text{…},X_{J};\hat{\Omega },\hat{\phi })$ for each of the N observations. In Appendix A we show that this estimator approaches the marginal maximum likelihood estimator as M→∞ if $\hat{\Omega }$ approaches the marginal maximum likelihood estimator (i.e., if K→∞). Below we show that with M=1, these estimates are reasonably accurate in a small secondary simulation conducted for just this purpose. We also use this method to estimate factor correlations in a real data application.

We simulate 50 datasets of 10,000 observations from a 10D MIRT model, where half of the observations are missing. All conditions are kept equal to the simulation study in Section 5, except for the fact that the true ability values are drawn from a multivariate normal distribution, where all correlations are set to 0.4. We estimate parameters using MML and a CVAE with 25 importance weights. Based on pilot studies, we found that MHRM provided better slope parameters when factors were correlated, whereas quasi‐Monte Carlo EM (QMCEM) provides better intercept estimates. Therefore, we include both estimation methods in this simulation.

Table 4 provides the recovery statistics for the different parameters. First of all, results indicate that the CVAE provides the most accurate ability estimates, which is in line with the results of the uncorrelated simulation study. Surprisingly, MML using MHRM actually results in clearly worse intercept estimates than the other two methods, whereas QMCEM performs clearly worse on the slopes, indicating that both MML methods struggle to obtain accurate item parameters on high‐dimensional correlated latent factors, when a large proportion of data is missing. In terms of the factor correlation estimates, the two MML methods are both more accurate than the CVAE. Overall the results show that VAE‐based methods are also applicable in situations where factors are correlated, although the factor correlation estimates themselves might be more precise with MML.

**TABLE 4:** Mean squared error of parameter estimates for correlated latent factors. Standard errors are reported between brackets. a denotes slope parameters, b intercept parameters, θ ability estimates, and r factor correlations.

	a			b			θ			r		
Model	$\sigma^{2}$	$bias^{2}$	mse	$\sigma^{2}$	$bias^{2}$	mse	$\sigma^{2}$	$bias^{2}$	mse	$\sigma^{2}$	$bias^{2}$	mse
CVAE	.0001	.0024	.0025	.0001	.0070	.0071	.0095	.3820	.392	<.0001	.0748	.0748
MHRM	.0051	.0006	.0056	.0061	.0128	.0189	.1970	.3470	.544	.0004	.0065	.0069
QMCEM	.0250	.0199	.0449	.0061	.0004	.0065	.1930	.3570	.550	.0161	.0277	.0438

## 8 REAL DATA APPLICATION

To demonstrate the practical viability of the different missing‐data approaches, we apply our models to a subset of the Bridge to Algebra (BTA) dataset, which was initially published in the context of the 2010 Association for Computing Machinery (ACM) Knowledge Discovery and Data‐Mining (KDD) competition (Stamper & Pardos, 2016). The dataset is publicly available in the Pittsburgh Science of Learning Center Datashop (Koedinger et al., 2010) and was gathered from an online environment for learning mathematics called the cognitive tutor (Ritter et al., 2007). The complete BTA dataset consists of responses of 6034 students to over 50,000 algebra problems. Importantly, each problem is divided into several steps, where each step is assumed to require one or more algebra skills. The full item set measures 60 different algebra skills. These skills consist of basic arithmetic operations, such as ‘identifying number as a common factor’ or ‘calculating zero partial product’. In our study, we treat the individual steps as items. The skill requirements for each item make the data well suited for MIRT as the skills can be seen as latent dimensions and the dataset already describes which items require which skills, effectively describing a large Q‐matrix.

To demonstrate the use of our approach, we selected 75 items which measure nine skills in total. Table 5 contains the descriptions of the latent dimensions as well as the number of items that load on each dimension. 1289 students completed this set of items. All students completed all 75 items, which allowed us to introduce missing data artificially and compare results between the complete and missing‐data datasets. The preprocessed dataset is available on GitHub.

**TABLE 5:** Number of items per latent dimension.

Latent dim.	Number of items
List consecutive multiples of a number	16
Calculate zero partial product	16
Identify number as common factor	12
Calculate partial product – carry out	16
Identify number as common multiple	16
List factor of large number	8
Calculate partial product – carry in	13
Calculate partial product – carry in and out	12
Calculate partial product – no carry	23

As a gold standard, we fit a nine‐dimensional M2PL model to the complete dataset using MML. Slopes were fit to zero based on the skill requirement data from the BTA dataset. Most items load on a single latent dimension, but some load on up to five dimensions. Each latent dimension is measured by at least eight items, and at most 23 items. The complete matrix of skill requirements is available on GitHub. To compare the approaches under missing data, we introduced 30% missing values at random. Parameters were estimated using both MML and the CVAE. We used 25 IW samples. Other hyperparameters were kept equal to the simulation study.

**FIGURE 2:**
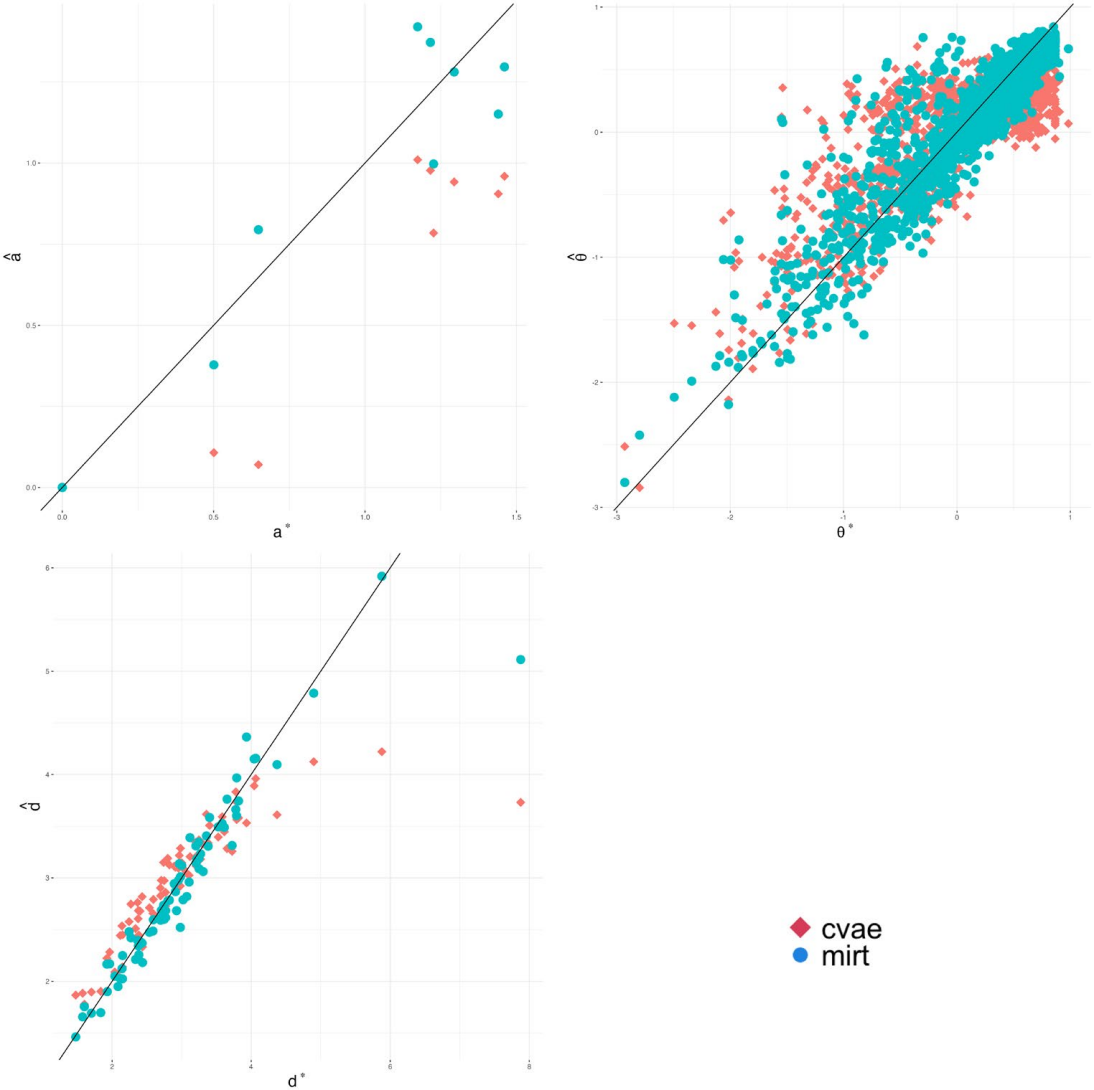
MIRT and CVAE parameters for 30% missing data, plotted against the MIRT parameter estimates based on the complete dataset.

Parameter estimation on the missing‐data dataset took 22 min using MML and just 16 s using the CVAE, highlighting the computational efficiency of variational methods. Figure 2 plots the parameter estimates on missing data against the parameter estimates on the entire dataset. Note that we only show the ability and discrimination parameters for a single dimension, which we think is representative of the other dimensions. The complete set of plots for all nine dimensions is available on GitHub, and additionally Table 6 reports the correlation coefficients between the missing data parameter estimates and the parameter estimates on the entire dataset for all dimensions. Overall, we see that MML estimates on the dataset with missing data are more similar to the full‐data MML estimates. This is in line with expectations, as these parameters are estimated using the same method with slightly different data. However, CVAE estimates also correlate highly with the older standard.

**TABLE 6:** Correlation between CVAE and MML parameter estimates with gold standard for each dimension. a denotes slopes, b intercepts, θ abilities, and r factor correlations between each latent factor and the eight other factors.

Latent dim.	1	2	3	4	5	6	7	8	9
$a_{\mathrm{cvae}}$	.9291	.9734	.9567	.9151	.9696	.9714	.5003	.7368	.6028
$a_{\mathrm{mml}}$	.9424	.9469	.9917	.9810	.9968	.9856	.8240	.8540	.8967
$\theta_{\mathrm{cvae}}$	.7496	.7465	.8618	.8000	.8356	.7828	.3680	.3792	.7685
$\theta_{\mathrm{mml}}$	.8310	.8375	.9319	.9150	.9149	.9000	.8171	.8373	.8904
$r_{\mathrm{cvae}}$	.8296	.8245	.9603	.8090	.9493	.8993	.7583	.8838	.8676
$r_{\mathrm{mml}}$	.9455	.9606	.9963	.9730	.9728	.9846	.8864	.9695	.9257
$b_{\mathrm{cvae}}$	.8384	–	–	–	–	–	–	–	–
$b_{\mathrm{mml}}$	.9356	–	–	–	–	–	–	–	–

## 9 DISCUSSION

We studied four different variational methods of estimating M2PL models in the presence of missing data. In a simulation study, we compared the different VAE methods to the performance of MML estimation. Results confirm that variational methods are an efficient alternative to MML for estimating high‐dimensional MIRT models given that enough IW samples are used to attain performance up to par with MML. In situations where little to no data are missing, taking a small number of importance samples in the VAE‐based models is already sufficient for a performance comparable to MML. However, when large parts of the data are not available, which might occur in adaptive testing settings or test equating environments for example, the ELBO appears to underestimate the marginal log‐likelihood, and more IW samples will be needed to accurately recover the model parameters. In terms of the different variational methods used, it is clear that the CVAE and IMVAE should be preferred to the straightforward input‐dropout method, which has been used in the context of VAE‐based MIRT (Liu et al., 2022). Imputing missing values with 0 leads to suboptimal ability estimates, which in turn affects the estimation of the item parameters. This is most relevant if a substantial portion of data is missing. In terms of parameter recovery accuracy, the CVAE is the best model, as it provides the inference model with extra information regarding missing values, enabling the model to deal with missing values correctly. The IMVAE might be used as a simpler alternative, sacrificing some estimation accuracy for a less complex model. Finally, we have shown that the CVAE can provide accurate parameter estimates on real high‐dimensional datasets over 80 times faster than traditional MML.

One limitation of our study concerns the missing data model. In the current simulations, as well as the real data application, we assumed that all data were missing completely at random (MCAR). This simplifying assumption allowed us to make a general comparison between different approaches to handling missing data. Our model approaches full‐information marginal maximum likelihood as the number of samples increases, so we expect the present approach to be viable in the case of missing at random (MAR) as well. However, in future work, it will be important to verify whether our proposed methods generalize to situations where data are MAR.

## AUTHOR CONTRIBUTIONS

**Karel Veldkamp:** conceptualization; investigation; writing – original draft; methodology; validation; visualization; software; data curation; formal analysis; project administration. **Raoul Grasman:** conceptualization; investigation; writing – review and editing; methodology; validation; formal analysis; supervision; project administration. **Dylan Molenaar:** supervision; writing – review and editing; validation; methodology; investigation; conceptualization; formal analysis; project administration.

## CONFLICT OF INTEREST STATEMENT

The authors declare that they have no conflicts of interest.

## Data Availability

All data that support the findings of this study are available at https://github.com/KarelVeldkamp/VAE‐MIRT‐Missing. These data were derived from the following resources available in the public domain: – PLSC Datashop: https://pslcdatashop.web.cmu.edu/DatasetInfo?datasetId=4524. All code required to reproduce our experiments is also available on GitHub: https://github.com/KarelVeldkamp/VAE‐MIRT‐Missing

## Footnotes

^1^
This concerns the classification of objects based on unordered observed points in 3D space.

^2^
We thank the anonymous reviewer who brought this dissertation to our attention.

^3^

https://github.com/KarelVeldkamp/VAE‐MIRT‐Missing.

^4^
Since the main simulation study was run on a cluster computer, where different simulation runs are executed on different computational nodes, we performed a separate 10‐iteration study locally solely for the purpose of fairly comparing runtimes.

## APPENDIX A DERIVING THE MML ESTIMATOR OF THE COVARIANCE MATRIX

The marginal likelihood of a set of responses ${\left\{x_{ij}\right\}}_{j=1}^{J}$ for person i is

$$L_{i}=\int d\theta f(\theta ;\Sigma )\overset{J}{\underset{j=1}{\prod }}P(X_{ij}=x_{ij}|\Omega_{j},\theta ),$$

where f(θ;Σ) is the multivariate joint normal density, given a mean vector μ=0 and a covariance matrix Σ, which is d×d:

$$f(\theta )=\frac{1}{{\left(2\pi \right)}^{d/2}|\Sigma {|}^{1/2}}\exp\left(-\frac{1}{2}\theta^{\prime }\Sigma^{-1}\theta \right).$$

The derivative of $\log L_{i}$ with respect to Σ is

$$\begin{array}{l} \frac{\partial }{\partial \Sigma }\log L_{i}=\frac{1}{L_{i}}\frac{\partial }{\partial \Sigma }L_{i}\\ =\frac{1}{L_{i}}\int d\theta \frac{\partial f}{\partial \Sigma }(\theta ;\Sigma )P(X_{1},\text{…},X_{J}|\Omega ,\theta_{i}=\theta ). \end{array}$$

Logarithmic differentiation (dg/dy=g(y)(dlogg/dy)) gives

$$\begin{array}{ll} \frac{\partial f}{\partial \Sigma } & =f(\theta ;\Sigma )\frac{\partial }{\partial \Sigma }[-\frac{d}{2}\log|\Sigma |-\frac{d}{2}\theta^{\prime }\Sigma^{-1}\theta ]\;\\ & =f(\theta ;\Sigma )[-\frac{d}{2}\Sigma^{-1}+\frac{d}{2}\Sigma^{-1}\theta \theta^{\prime }\Sigma^{-1}].\; \end{array}$$

Therefore,

$$\frac{\partial }{\partial \Sigma }\log L_{i}=\frac{1}{L_{i}}\int d\theta f(\theta ;\Sigma )\frac{d}{2}[-\Sigma^{-1}+\Sigma^{-1}\theta \theta^{\prime }\Sigma^{-1}]P(X_{1},\text{…},X_{J}|\Omega ,\theta_{i}=\theta ).$$

Note that

$$\int d\theta f(\theta ;\Sigma )[-\frac{d}{2}\Sigma^{-1}]P(X_{1},\text{…},X_{J}|\Omega ,\theta_{i}=\theta )=-\frac{d}{2}\Sigma^{-1}L_{i}$$

and

Therefore,

To find the marginal maximum likelihood (MML) estimator of Σ, we equate the derivative of the sample log‐likelihood to zero: Summing over i=1,…,N, we have

$$\begin{array}{c} \frac{\partial }{\partial \Sigma }\log L=0\;\Leftrightarrow \;N\Sigma^{-1}=\Sigma^{-1}\left[\overset{N}{\underset{i=1}{\sum }}\frac{1}{L_{i}}\int d\theta f(\theta ;\Sigma )\theta \theta^{\prime }P(X_{1},\text{…},X_{J}|\Omega ,\theta_{i}=\theta )\right]\Sigma^{-1}\;\;\\ \Leftrightarrow \;\Sigma =\frac{1}{N}\overset{n}{\underset{i=1}{\sum }}\int \theta \theta^{\prime }\frac{1}{L_{i}}f(\theta ;\Sigma )P(X_{1},\text{…},X_{J}|\Omega ,\theta_{i}=\theta )d\theta .\; \end{array}$$

Consequently, using Bayes' theorem,

$$∴\Sigma =\frac{1}{N}\overset{N}{\underset{i=1}{\sum }}\int \theta \theta^{\prime }f(\theta |X_{1}=x_{i1},\text{…},X_{J}=x_{iJ};\Omega ,\Sigma )d\theta .$$

That is, the MML estimator of the covariance matrix satisfies the equation

$$\Sigma =\frac{1}{N}\overset{N}{\underset{i=1}{\sum }}E\{\theta \theta^{\prime }|X_{1}=x_{i1},\text{…},X_{J}=x_{iJ};\Omega ,\Sigma \}.$$

Note that this is in fact a sample implementation of the equation $E\{\eta \eta^{\prime }\}=E\{E\{\eta \eta^{\prime }|\text{x}\}\}$. In other words, the MML estimator of Σ solves a *method of moments* estimator equation.

Note that if we can sample from the posterior for each subject i, yielding $\eta_{i},i=1,\text{…},n$, we can obtain an estimate of Σ:

$$\hat{\Sigma }=\frac{1}{N}\overset{N}{\underset{i=1}{\sum }}\eta_{i}\eta_{i}^{\prime },\;\eta_{i}∼f(\theta |X_{1}=x_{i1},\text{…},X_{J}=x_{iJ};\Omega ,\Sigma ).$$

If we average many such estimates, repeatedly sampling from the posteriors, this estimate converges in probability to the MML estimate if $\Omega =\hat{\Omega }$ the MML estimate.

## References

[bmsp12363-bib-0001] Amari, S.‐i. (1993). Backpropagation and stochastic gradient descent method. Neurocomputing, 5(4–5), 185–196.

[bmsp12363-bib-0002] Bergner, Y. , Halpin, P. , & Vie, J.‐J. (2022). Multidimensional item response theory in the style of collaborative filtering. Psychometrika, 87(1), 266–288. 34698979 10.1007/s11336-021-09788-9

[bmsp12363-bib-0003] Blei, D. M. , Kucukelbir, A. , & McAuliffe, J. D. (2017). Variational inference: A review for statisticians. Journal of the American Statistical Association, 112(518), 859–877.

[bmsp12363-bib-0004] Bock, R. D. , & Aitkin, M. (1981). Marginal maximum likelihood estimation of item parameters: Application of an em algorithm. Psychometrika, 46(4), 443–459.

[bmsp12363-bib-0005] Browne, M. W. (1974). Generalized least squares estimators in the analysis of covariance structures. South African Statistical Journal, 8(1), 1–24.

[bmsp12363-bib-0006] Burda, Y. , Grosse, R. , & Salakhutdinov, R. (2015). Importance weighted autoencoders. *arXiv preprint arXiv:1509.00519*.

[bmsp12363-bib-0007] Cai, L. (2010). High‐dimensional exploratory item factor analysis by a Metropolis–Hastings Robbins–Monro Algorithm. Psychometrika, 75, 33–57.

[bmsp12363-bib-0008] Chalmers, R. P. (2012). MIRT: A multidimensional item response theory package for the r environment. Journal of Statistical Software, 48, 1–29.

[bmsp12363-bib-0009] Chen, Y. , Li, X. , & Zhang, S. (2019). Joint maximum likelihood estimation for high‐dimensional exploratory item factor analysis. Psychometrika, 84, 124–146. 30456747 10.1007/s11336-018-9646-5

[bmsp12363-bib-0010] Cho, A. E. , Wang, C. , Zhang, X. , & Xu, G. (2021). Gaussian variational estimation for multidimensional item response theory. British Journal of Mathematical and Statistical Psychology, 74, 52–85. 33064318 10.1111/bmsp.12219

[bmsp12363-bib-0011] Collier, M. , Nazabal, A. , & Williams, C. K. (2020). VAEs in the presence of missing data. *arXiv preprint arXiv:2006.05301*.

[bmsp12363-bib-0012] Converse, G. , Curi, M. , Oliveira, S. , & Templin, J. (2021). Estimation of multidimensional item response theory models with correlated latent variables using variational autoencoders. Machine Learning, 110(6), 1463–1480.

[bmsp12363-bib-0013] Cremer, C. , Morris, Q. , & Duvenaud, D. (2017). Reinterpreting importance‐weighted autoencoders. *arXiv preprint arXiv:1704.02916*.

[bmsp12363-bib-0014] Curi, M. , Converse, G. A. , Hajewski, J. , & Oliveira, S. (2019). Interpretable variational autoencoders for cognitive models. In 2019 International Joint Conference on Neural Networks (IJCNN) (pp. 1–8).

[bmsp12363-bib-0015] da Silva, M. A. , Liu, R. , Huggins‐Manley, A. C. , & Bazán, J. L. (2019). Incorporating the Q‐matrix into multidimensional item response theory models. Educational and Psychological Measurement, 79(4), 665–687. 32655178 10.1177/0013164418814898 PMC7328237

[bmsp12363-bib-0016] Edwards, M. C. (2010). A Markov chain Monte Carlo approach to confirmatory item factor analysis. Psychometrika, 75(3), 474–497.

[bmsp12363-bib-0017] Hornik, K. , Stinchcombe, M. , & White, H. (1989). Multilayer feedforward networks are universal approximators. Neural Networks, 2(5), 359–366.

[bmsp12363-bib-0018] Kingma, D. P. , & Welling, M. (2013). Auto‐encoding variational bayes. *arXiv preprint arXiv:1312.6114*.

[bmsp12363-bib-0019] Klinkenberg, S. , Straatemeier, M. , & van der Maas, H. L. (2011). Computer adaptive practice of maths ability using a new item response model for on the fly ability and difficulty estimation. Computers & Education, 57(2), 1813–1824.

[bmsp12363-bib-0020] Koedinger, K. R. , Baker, R. S. , Cunningham, K. , Skogsholm, A. , Leber, B. , & Stamper, J. (2010). A data repository for the EDM community: The PSLC DataShop. Handbook of Educational Data Mining, 43, 43–56.

[bmsp12363-bib-0021] Liu, T. , Wang, C. , & Xu, G. (2022). Estimating three‐and four‐parameter mirt models with importance‐weighted sampling enhanced variational auto‐encoder. Frontiers in Psychology, 13, 4189. 10.3389/fpsyg.2022.935419 PMC9421264 36046415

[bmsp12363-bib-0022] Lord, F. M. , & Novick, M. R. (1968). Statistical theories of mental test scores. IAP.

[bmsp12363-bib-0023] Ma, C. , Gong, W. , Hernández‐Lobato, J. M. , Koenigstein, N. , Nowozin, S. , & Zhang, C. (2018). Partial VAE for hybrid recommender system. In NIPS workshop on Bayesian deep learning (Vol. 2018). NIPS.

[bmsp12363-bib-0024] Ma, C. , Ouyang, J. , Wang, C. , & Xu, G. (2023). A note on improving variational estimation for multidimensional item response theory. Psychometrika, 89(1), 172–204. 37979074 10.1007/s11336-023-09939-0

[bmsp12363-bib-0025] McKinley, R. L. , & Reckase, M. D. (1983). An extension of the two‐parameter logistic model to the multidimensional latent space. (Tech. Rep.). American Coll Testing Program Iowa City Ia Resident Programs Dept.

[bmsp12363-bib-0026] Meyer, J. P. , & Zhu, S. (2013). Fair and equitable measurement of student learning in MOOCS: An introduction to item response theory, scale linking, and score equating. Research & Practice in Assessment, 8, 26–39.

[bmsp12363-bib-0027] Montecino, C. E. E. (2023). Using VAE for incomplete educational data (Unpublished doctoral dissertation). Universidade de São Paulo.

[bmsp12363-bib-0028] Muthén, B. (1984). A general structural equation model with dichotomous, ordered categorical, and continuous latent variable indicators. Psychometrika, 49(1), 115–132.

[bmsp12363-bib-0029] Nazabal, A. , Olmos, P. M. , Ghahramani, Z. , & Valera, I. (2020). Handling incomplete heterogeneous data using VAEs. Pattern Recognition, 107, 107501.

[bmsp12363-bib-0030] Oka, M. , Chen, Y. , & Mounstaki, I. (2024). Learning high‐dimensional latent variable models via doubly stochastic optimisation by unadjusted langevin. *arXiv preprint arXiv:2406.09311*.

[bmsp12363-bib-0031] Paszke, A. , Gross, S. , Massa, F. , Lerer, A. , Bradbury, J. , Chanan, G. , Killeen, T. , Lin, Z. , Gimelshein, N. , Antiga, L. , Desmaison, A. , Kopf, A. , Yang, E. , DeVito, Z. , Raison, M. , Tejani, A. , Chilamkurthy, S. , Steiner, B. , Fang, L. , … Chintala, S. (2019). Pytorch: An imperative style, high‐performance deep learning library. In Advances in neural information processing systems 32 (pp. 8024–8035). Curran Associates, Inc. http://papers.neurips.cc/paper/9015‐pytorch‐an‐imperative‐style‐high‐performance‐deep‐learning‐library.pdf

[bmsp12363-bib-0032] Qi, C. R. , Su, H. , Mo, K. , & Guibas, L. J. (2017). Pointnet: Deep learning on point sets for 3d classification and segmentation. In Proceedings of the IEEE conference on computer vision and pattern recognition (pp. 652–660).

[bmsp12363-bib-0033] Reckase, M. D. (2009). Multidimensional item response theory models. Springer.

[bmsp12363-bib-0034] Reddi, S. J. , Kale, S. , & Kumar, S. (2019). On the convergence of adam and beyond. *arXiv preprint arXiv:1904.09237*.

[bmsp12363-bib-0035] Rezende, D. , & Mohamed, S. (2015). Variational inference with normalizing flows. In International conference on machine learning (pp. 1530–1538).

[bmsp12363-bib-0036] Ritter, S. , Anderson, J. R. , Koedinger, K. R. , & Corbett, A. (2007). Cognitive tutor: Applied research in mathematics education. Psychonomic Bulletin & Review, 14, 249–255. 17694909 10.3758/bf03194060

[bmsp12363-bib-0037] Schilling, S. , & Bock, R. D. (2005). High‐dimensional maximum marginal likelihood item factor analysis by adaptive quadrature. Psychometrika, 70, 533–555.

[bmsp12363-bib-0038] Stamper, J. , & Pardos, Z. A. (2016). The 2010 KDD Cup Competition dataset: Engaging the machine learning community in predictive learning analytics. Journal of Learning Analytics, 3(2), 312–316.

[bmsp12363-bib-0039] Svozil, D. , Kvasnicka, V. , & Pospichal, J. (1997). Introduction to multi‐layer feed‐forward neural networks. Chemometrics and Intelligent Laboratory Systems, 39(1), 43–62.

[bmsp12363-bib-0040] Takane, Y. , & De Leeuw, J. (1987). On the relationship between item response theory and factor analysis of discretized variables. Psychometrika, 52(3), 393–408.

[bmsp12363-bib-0041] Thomas, M. L. (2019). Advances in applications of item response theory to clinical assessment. Psychological Assessment, 31(12), 1442. 30869966 10.1037/pas0000597 PMC6745011

[bmsp12363-bib-0042] Urban, C. J. , & Bauer, D. J. (2021). A deep learning algorithm for high‐dimensional exploratory item factor analysis. Psychometrika, 86(1), 1–29. 33528784 10.1007/s11336-021-09748-3

[bmsp12363-bib-0043] von Davier, M. , & Sinharay, S. (2010). Stochastic approximation methods for latent regression item response models. Journal of Educational and Behavioral Statistics, 35(2), 174–193.

[bmsp12363-bib-0044] Wirth, R. , & Edwards, M. C. (2007). Item factor analysis: Current approaches and future directions. Psychological Methods, 12(1), 58. 17402812 10.1037/1082-989X.12.1.58 PMC3162326

[bmsp12363-bib-0045] Wood, R. , Wilson, D. , Gibbons, R. , Schilling, S. , Muraki, E. , & Bock, R. (2002). Testfact: Test scoring, item statistics, and item factor analysis. Chicago. Scientific Software International Inc.

[bmsp12363-bib-0046] Wu, M. , Davis, R. L. , Domingue, B. W. , Piech, C. , & Goodman, N. (2020). Variational item response theory: Fast, accurate, and expressive. *arXiv preprint arXiv:2002.00276*.

[bmsp12363-bib-0047] Zhang, C. , Bütepage, J. , Kjellström, H. , & Mandt, S. (2018). Advances in variational inference. IEEE Transactions on Pattern Analysis and Machine Intelligence, 41(8), 2008–2026. 30596568 10.1109/TPAMI.2018.2889774

[bmsp12363-bib-0048] Zhang, S. , & Chen, Y. (2022). Computation for latent variable model estimation: A unified stochastic proximal framework. Psychometrika, 87(4), 1473–1502. 35524934 10.1007/s11336-022-09863-9 PMC9636119

[bmsp12363-bib-0049] Zhang, S. , Chen, Y. , & Liu, Y. (2020). An improved stochastic em algorithm for large‐scale full‐information item factor analysis. British Journal of Mathematical and Statistical Psychology, 73(1), 44–71. 30511445 10.1111/bmsp.12153

